# Concomitant Prostate Cancer and Hodgkin Lymphoma: A Differential Diagnosis Guided by a Combined 68Ga-PSMA-11 and 18F-FDG PET/CT Approach

**DOI:** 10.3390/medicina57090975

**Published:** 2021-09-17

**Authors:** Alberto Miceli, Mattia Riondato, Francesca D’Amico, Maria Isabella Donegani, Nataniele Piol, Marco Mora, Bruno Spina, Silvia Morbelli, Matteo Bauckneht

**Affiliations:** 1 Department of Health Sciences (DISSAL), University of Genova, 16132 Genova, Italy; albertomiceli23@gmail.com (A.M.); damicofrancesca@outlook.com (F.D.); isabella.donegani@gmail.com (M.I.D.); 2 Nuclear Medicine, IRCCS Ospedale Policlinico San Martino, 16132 Genova, Italy; mattia.riondato@hsanmartino.it (M.R.); matteo.bauckneht@gmail.com (M.B.); 3 Division of Pathology, IRCCS Ospedale Policlinico San Martino, 16132 Genova, Italy; nataniele.piol@hsanmartino.it (N.P.); marco.mora@hsanmartino.it (M.M.); bruno.spina@hsanmartino.it (B.S.)

**Keywords:** PSMA PET/CT, FDG PET/CT, prostate cancer, Hodgkin lymphoma

## Abstract

Here we report the case of concomitant favorable-risk prostate cancer and Hodgkin Lymphoma in a 38-year old male. 68Ga-Prostate Specific Membrane Antigen-11 Positron Emission Tomography/Computed Tomography (68Ga-PSMA-11 PET/CT) was performed for staging purposes, showing the focal PSMA prostatic uptake as well as the presence of enlarged low-PSMA expressing mediastinal lymphadenopathies, thus raising the suspicion of another malignancy. A subsequent 18F-Fluorodeoxyglucose (18F-FDG) PET/CT demonstrated a high FDG-avidity by mediastinal lymphadenopathies as opposed to the low prostate cancer FDG uptake. Of note, both tumor entities were clearly detected by the two scans. However, different ranges in terms of Maximum Standardized Uptake Value (SUVmax) uptake allowed the discrimination between the two tumor entities. At the subsequent mediastinal lymph nodal biopsy, the coexistence of Hodgkin lymphoma was documented. The present case suggests that even if specific for prostate cancer, 68Ga-PSMA-11 PET/CT may raise the suspicion of other concurrent malignancies thanks to its non-receptor bounding mechanism. Further, it shows that in certain cases, the combination of 18F-FDG and 68Ga-PSMA PET/CT imaging may non-invasively guide the clinical management, optimizing the diagnostic process and the subsequent therapeutic interventions.

## 1. Case Report

**Figure 1:**
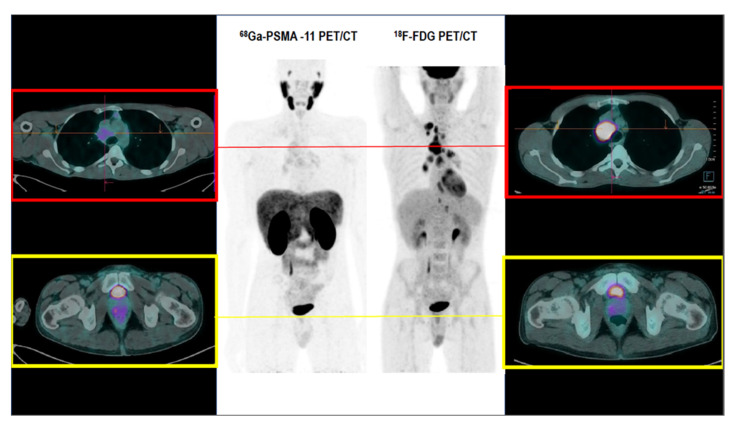
The central panel displays the Maximum Intensity Projection (MIP) of 68Ga-PSMA-11 and 18F-FDG PET/CT scans. The left axial hybrid PET/CT images show the focal 68Ga-PSMA uptake at the peripheral zone of the right prostatic lobe (**yellow**) and the mild tracer uptake by enlarged mediastinal lymph nodes (**red**). The right axial hybrid PET/CT images show the high 18F-FDG uptake by the enlarged mediastinal lymphatic stations (**red**) and the low 18F-FDG uptake by the peripheral zone of the right prostatic lobe (**yellow**).

A 38-year-old man was referred to our Institution in May 2020 for the diagnostic workup for weight loss, asthenia, and Prostate-Specific Antigen (PSA) elevation (10.49 ng/mL). A right lobe prostatic adenocarcinoma (Gleason score 3 + 3) was diagnosed after pelvic Magnetic Resonance Imaging (MRI) and fusion biopsy. Given the favorable risk, the active surveillance strategy was started [1,2]. However, given the subsequent raising of PSA (13.45 ng/mL), and the appearance of an enlarged right supraclavicular lymphadenopathy, the patient underwent a 68Ga-Prostate Specific Membrane Antigen-11 Positron Emission Tomography/Computed Tomography (68Ga-PSMA-11 PET/CT) for staging purposes. The 68Ga-PSMA-11 PET/CT scan documented a moderate focal 68Ga-PSMA-11 uptake in the right lobe of the prostate (Maximum Standardized Uptake Value, SUVmax 7.2, Figure 1), coherent with known primary prostate cancer. However, enlarged low-PSMA expressing mediastinal lymphadenopathies were also observed (SUVmax 5.1, Figure 1). The coexistence of moderate PSMA expressing primary prostate carcinoma and low PSMA expressing enlarged lymphadenopathies might be misinterpreted as due to the same underlying histopathology. However, given the favourable risk profile of prostate carcinoma and the atypical site of nodal disease, the suspicion of another malignancy independently involving mediastinal lymph nodes was raised. A subsequent 18F-Fluorodeoxyglucose (18F-FDG) PET/CT was thus performed, in order to guide the histopathological sampling and for staging purposes. 18F-FDG PET/CT showed high tracer uptake by multiple mediastinal lymphadenopathies (SUVmax 15.3 at the station 4R, Figure 1), paralleled by the low right lobe prostate cancer 18F-FDG-avidity (SUVmax 3.5, Figure 1). According to 18F-FDG PET/CT findings, ultrasound-guided transbronchial needle aspiration was performed, showing the presence of a concomitant Hodgkin lymphoma, scleronodular variant. Bleomycin-Dacarbazine-Doxorubicin-Vinblastine (ABVD) chemotherapy scheme was thus administered to the patient obtaining complete metabolic response (CMR—Lugano Classification) at interim as well as at the end-of-therapy 18F-FDG PET/CT [3]. Prostate cancer active surveillance is currently ongoing.

## 2. Discussion

The present case represents an excellent demonstration of lesion characterization using molecular imaging, as the two different PET-tracers provided complementary information able to guide the diagnostic workup leading to a prognosis-priority treatment plan. PET/CT imaging with PSMA-labelled radiotracers is influencing more and more the clinical practice in the initial staging of prostate cancer, given its superior sensitivity, specificity and diagnostic accuracy compared to the standard of care (bone scan and CT) and compared to other PET tracers (i.e., choline) [4,5,6]. However, in addition to the specificity of these tracers for the prostatic tissues, PSMA molecules can be concentrated even by a wide range of solid and haematologic malignancies due to its expression by the cancer neovascularization, in the absence of a specific receptor-mediated mechanism [7,8,9]. Conversely, in contrast with the advanced metastatic hormone-refractory stage [10,11,12], the low 18F-FDG-avidity of well-differentiated, low-grade, naïve prostate cancer makes this tool not routinely recommended in this clinical setting [13]. The divergent uptake pattern of the two radiotracers thus raised the suspicion of the coexistence of two different tumour entities, which were subsequently pathologically confirmed. A failure to consider uptake by a non-prostatic malignancy on 68Ga-PSMA-11 PET/CT scan could potentially lead to scan misinterpretation as metastatic prostate carcinoma. Other alternative diagnoses should always be ruled out when 68Ga-PSMA-11 PET/CT identifies nodal disease at an atypical site. 

Only a few cases available in the literature previously described a combined 68Ga-PSMA-11 and 18F-FDG PET/CT approach in patients with concurrent prostate cancer and follicular lymphoma [9,14,15,16]. However, this is the first case in which a similar approach led to the final diagnosis of concurrent Hodgkin lymphoma. Hodgkin lymphoma should thus be added to the (already long) list of differential diagnosis to be considered when low-PSMA avid lymph nodes are observed in an atypical site in patients with prostate carcinoma.

## 3. Conclusions

Even if specific for prostate cancer, 68Ga-PSMA-11 PET/CT may raise the suspicion of other con-current malignancies thanks to its non-receptor bounding mechanism. In certain cases, the combination of 18F-FDG and 68Ga-PSMA-11 PET/CT imaging may non-invasively guide the clinical management, optimizing the diagnostic process and the subsequent therapeutic interventions.

## Data Availability

Not applicable.
